# 783. Lung Transplantation for COVID-19-Associated Lung Injury (CALI): An International Registry

**DOI:** 10.1093/ofid/ofac492.044

**Published:** 2022-12-15

**Authors:** Maha M Alamri, Rebecca Osborn, Reda Girgis, Nicholas Marschalk, Ryan Rivosecchi, Michael G Ison, Max Weder, Saima Aslam

**Affiliations:** Northwestern Memorial Hospital, Chicago, Illinois; Northwestern Memorial Hospital, Chicago, Illinois; Spectrum Health, Grand Rapids, Michigan; Ohio State University, Columbus, Ohio; University of Pittsburgh, Pittsburgh, Pennsylvania; Northwestern University Feinberg School of Medicine, CHICAGO, Illinois; University of Virginia, Charlottesville, Virginia; Univesity of California at San Diego, San Diego, California

## Abstract

**Background:**

SARS-CoV-2 can result in a range of infections from asymptomatic disease to progressive COVID-19 and death. In some pts with CALI, lung transplantation (LTx) may be lifesaving. Up to 10% of LTx in the US is currently for pts with CALI. Understanding the characteristics and outcomes of these pts is critical.

**Methods:**

A open-access electronic registry was established to collect de-identified data from pts who have undergone LTx for CALI from centers globally. The study was IRB approved at Northwestern with a wavier for consent (no PHI is collected sites could submit data about pre-Tx, peri-Tx and post-Tx course). Follow-up for 1-yr post-LTx was collected.

**Results:**

To date, 89 pts with complete day 30 post-LTx data have been entered into the registry. Pt demographics and pre-Tx status are shown in Table 1. 3 pts required oxygen prior to COVID-19. Most sites required neg PCR tests prior to listing (11 (12.4%) required no - PCRs, 11 (12.4%) required 1 and 61 (68.5%) required 2). LTx occurred 137 days post-infection and none developed COVID-19 in the first 30 d; 4 were given monoclonal antibodies post-tx. Post-tx ICU LOS averaged 24.5 d with total post-tx hospitalization of 37.6 d (See Table 2). Most experienced infectious and non-infectious morbidity. Most (47.8%) required an additional 30 days of rehab. 2 pts died within 30 days due to sepsis and anoxia. 5 died between day 30 and 90 and an additional 12 died between day 90 and 365.

**Conclusion:**

The contribution of cases to this international registry is ongoing. While outcomes of LTx for CALI are generally good, patients experience prolonged post-transplant hospitalization, rehabilitation and significant morbidity and infections are common.

**Disclosures:**

**Michael G. Ison, MD MS**, GlaxoSmithKline: Advisor/Consultant|GlaxoSmithKline: Grant/Research Support **Saima Aslam, MD**, Armata: Grant/Research Support|BioMx: Advisor/Consultant|Contrafect: Grant/Research Support|Gilead: Honoraria|Merck: Honoraria|Phico: Advisor/Consultant.

